# Sequential Inoculation with Selected Indigenous Yeasts Enhances the Aroma Profiles and Typicity of White Wines from Yantai, China

**DOI:** 10.3390/foods14234015

**Published:** 2025-11-23

**Authors:** Zihao Zhai, Piaoran Zhang, Weidong Huang, Jicheng Zhan, Guangli Xia, Weifu Kong, Yilin You

**Affiliations:** 1 Beijing Key Laboratory of Viticulture and Enology, College of Food Science and Nutritional Engineering, China Agricultural University, Tsinghua East Road 17, Haidian District, Beijing 100083, China; 2 Yantai Institute of China Agricultural University, Binhai Middle Road 2006, Laishan District, Yantai 264670, China; 3 College of Pharmacy, Binzhou Medical University, uanhai Road 346, Laishan District, Yantai 264033, China; 4 Yantai Pula Valley Winery Management Co., Ltd., Yuangezhuang Village, Laishan District, Yantai 264000, China

**Keywords:** sequential inoculation, β-glucosidase, volatile compounds, terpenes, Yantai

## Abstract

The Yantai region in China, despite its ideal viticultural conditions, faces a challenge in producing white wines with distinct regional typicity. This study explores the potential of indigenous yeast strains—specifically, the *Hanseniaspora uvarum* H30 and *Saccharomyces cerevisiae* YT13, both isolated and identified from the Yantai vineyard environment—to enhance the aroma profiles and overall quality of white wines from this region. Italian Riesling and Petit Manseng grapes were fermented with two commercial *Saccharomyces cerevisiae* strains (EC1118 and Tropical/X16) and a sequential inoculation of the locally sourced *Hanseniaspora uvarum* H30 (with high β-glucosidase activity) followed by *S. cerevisiae* YT13. The results showed that sequential inoculation with H30 + YT13 significantly enhanced the concentration of key ester compounds and uniquely increased terpene levels. Notably, valuable terpenes such as farnesol and nerolidol were exclusively detected in the H30 + YT13 group for Italian Riesling. Sensory analysis confirmed that these chemical changes translated into higher scores for floral and fruity attributes in the sequentially inoculated wines. These findings demonstrate that sequential inoculation with indigenous yeasts effectively enhances the aromatic complexity and varietal typicity of white wines from Yantai, providing a promising strategy for developing wines with stronger regional identity.

## 1. Introduction

The variety and origin of grapes significantly influence their chemical and physical composition, which are key determinants of the final wine quality [1]. Taking white wines as an example, Italian Riesling produces light-bodied wines with bright acidity, exhibiting flavors of lime, green apple, and minerality, resulting in a crisp and straightforward palate [2]. In contrast, Petit Manseng—characterized by its high sugar and acid content—yields fuller-bodied wines with sharp acidity and a firm structure, showcasing ripe citrus, white peach, and tropical fruit notes, along with greater complexity and aging potential [3]. Additionally, different wine regions inevitably harbor distinct microbial communities due to varying climatic conditions, leading to diverse yeast populations with unique fermentation potentials that ultimately shape the final wine quality [4]. The Shandong Peninsula is China’s largest wine-producing region. Yantai, Shandong, boasts a prime geographical location, bordered by the Bohai and Yellow Seas, adjacent to Qingdao to the south, and facing Tianjin, Dalian, and the Korean Peninsula across the sea [5]. Its latitude is similar to that of Bordeaux, France, sharing a comparable maritime climate characterized by mild winters and the ideal “3S” conditions for viticulture: Sun, Sand, and Sea [6]. This makes it an exceptional terroir for wine grape cultivation, with Chardonnay, Italian Riesling, and Petit Manseng being the dominant white varieties.

Aroma compounds in grapes and wine exist in both free and bound forms. Typically, the free-form volatiles are sensorily perceptible due to their volatility, whereas the bound forms are non-volatile and thus function as aroma precursors. These bound precursors require hydrolysis to release their free volatile counterparts. The two most extensively studied types of bound aroma compounds are glycosidically bound and cysteine-bound conjugates. It has been found that glycosidically bound precursors are predominantly more abundant in grape berries than those bound to cysteine [7,8]. The release of these glycosidic aromas depends on β-glucosidase activity during fermentation [9]. β-Glucosidase, a type of cellulolytic enzyme, catalyzes the hydrolysis of glycosidic bonds in glycosidically bound aroma molecules, thereby releasing free volatile compounds. The aroma compounds released through β-glucosidase hydrolysis typically include monoterpenes, C13-norisoprenoids, and volatile phenols. Monoterpenes mainly consist of geraniol, nerol, linalool, linalool oxides, and some monoterpene polyols; C13-norisoprenoids include 3-hydroxy-β-damascone and α-ionol; and volatile phenols comprise benzyl alcohol, 2-phenylethanol, tyrosol, and zingerone [10,11,12]. However, β-glucosidase extracted from grapes is inhibited by alcohol [13], and the enzyme produced by *Saccharomyces cerevisiae* exhibits limited catalytic efficiency under fermentation conditions. In contrast, many non-*Saccharomyces* yeasts secrete β-glucosidase with higher activity, enhancing the release of glycosidic bound terpenes [14] and demonstrating better adaptability to winemaking environments [15]. Since non-*Saccharomyces* yeasts typically cannot complete fermentation alone, sequential inoculation with *S. cerevisiae* is a common and effective approach.

Although wild yeasts have been shown to contribute to regional wine characteristics, limited research has focused on the Yantai region. Despite its exceptional terroir and high-quality wines, Yantai lacks distinct regional typicity. To develop white wines with unique Yantai characteristics, this study sampled Italian Riesling and Petit Manseng grapes from the Pula Valley in Yantai and inoculated them with two commercial yeasts (EC1118 (Lallemand Inc., Montreal, QC, Canada) and X16 (Laffort, Floirac, France)/Tropical (Lamothe-Abiet, Canéjan, France)) as well as indigenous wild strains specifically isolated from the Yantai region: H30 (a non-*Saccharomyces* yeast, *Hanseniaspora uvarum*, with high β-glucosidase activity) and YT13 (a native *S. cerevisiae* strain with excellent fermentation performance) [16]. The H30 strain (*Hanseniaspora uvarum*) was originally isolated from a Chinese vineyard in the Yantai region and identified through molecular methods by Han [10]. It was subsequently selected as a high β-glucosidase-producing candidate via a primary screen using a pNPG colorimetric assay, followed by a secondary screen that confirmed its high enzymatic activity and specific activity compared to commercial yeast [10]. By analyzing basic physicochemical parameters, total phenols, phenolic acids, organic acids, volatile compounds, and sensory profiles of the resulting wines, this study investigates the impact of different yeasts on the quality of Yantai white wines, providing insights for crafting wines with regional distinctiveness.

## 2. Materials and Methods

### 2.1. Grape Processing and Fermentation Protocol

Grape processing involves several critical steps including harvesting, berry selection, destemming, and crushing [17]. During harvesting, mechanical contact between berries should be minimized to preserve fruit integrity, with diseased or rotten berries being rigorously removed. Must preparations employ pneumatic pressing, where significant pressure (typically 1.5–2.0 bar) is applied to achieve complete juice extraction from both pulp and skins, yielding approximately 70% free-run juice and 30% press juice. Potassium metabisulfite (100 mg/L) was added during pressing to: (1) inhibit oxidative browning and flavor deterioration [18], (2) suppress growth of undesirable microorganisms [19], and (3) enhance organic acid retention and volatile compound development. Simultaneously, pectinase (50 mg/L) was introduced to facilitate pectin hydrolysis and accelerate clarification [20]. The pressed juice was then cold settled at 4 °C for 24 h. Following racking, juices were transferred to fermentation vessels (500 mL glass bottles sealed with airlocks) at 90% capacity. All yeast strains were first activated in YPD liquid medium (cultured at 28 °C with shaking at 180 rpm for 24 h). Subsequently, a 10% (*v*/*v*) inoculum was transferred into a grape juice medium for pre-culture (under the same conditions of 28 °C and 180 rpm for 24 h), achieving a final cell density of 1 × 10^8^ CFU/mL. Each treatment group was inoculated (1% *v*/*v*) [16] with either commercial strains (EC1118 or Tropical/X16 as pure cultures) or wild yeasts (sequential fermentation of H30 followed by YT13 after 4 days), with experimental groups detailed in Table 1. Following inoculation, a colony concentration greater than 1 × 10^6^ CFU/mL in the grape juice was achieved, thereby securing a rapid initiation of the fermentation process. All fermentations were conducted in triplicate, with progress monitored through daily °Brix measurements. All fermentation groups were conducted at a controlled temperature range of 16–18 °C and completed fermentation by day 15.

### 2.2. Determination of Oenological Parameters

The pH of the wine after fermentation was measured using a calibrated pH meter. Fermentation progress was monitored through daily °Brix measurements using a temperature-compensated refractometer [21], while post-fermentation residual sugar content was quantified via wine analyzer in triplicate measurements. Comprehensive oenological parameters including total acidity (expressed as g/L tartaric acid equivalent), glycerol content (g/L), alcohol content (% *v*/*v*), and volatile acidity (g/L acetic acid equivalent) were determined using an automated wine analyzer (F17-WineScan FT120 Beijing Zhongxi Yuanda Technology Co., Ltd., Beijing, China) following OIV-compliant protocol. Organic acid profiles (malic, tartaric, citric, and lactic acids) were analyzed by HPLC (An XBridge^®^ C18 column (4.6 mm × 250 mm, 5 μm) was used; column temperature: 35 °C; detection: PDA at 210 nm; mobile phase: 0.1% phosphoric acid solution/methanol (97.5:2.5, *v*/*v*); flow rate: 0.8 mL/min; injection volume: 10 μL) according to Han [22].

### 2.3. Analysis of Phenolic Compounds

The phenolic composition of wines was comprehensively characterized through multiple analytical approaches. Total phenol content was determined spectrophotometrically using the Folin–Ciocalteu assay [23], with results expressed as gallic acid equivalents (GAE, mg/L). A standard curve was constructed with gallic acid solutions at concentrations of 5, 10, 20, 50, and 70 mg/L, yielding a linear regression equation (y = 0.0265x − 0.0641) with a high coefficient of determination (R^2^ = 0.997). For the measurement, 2.5 mL of a ten-fold diluted wine sample was mixed with 5 mL of Folin–Ciocalteu reagent and 10 mL of 7.5% sodium carbonate solution. The mixture was vortexed, incubated at 50 °C for 5 min, and its absorbance was measured at 760 nm against a blank. The flavonol content in wine was analyzed by HPLC using a gradient elution program with two mobile phases (A and B, both adjusted to pH 3.0) [24]. Quantification was achieved via an external standard method, using a calibration curve constructed from a mixed standard of eight flavonols with concentrations ranging from 1.0 to 10.0 mg/L. Prior to injection, the wine samples were prepared by direct filtration through a hydrophilic polyethersulfone syringe filter. The flavan-3-ol content in wine was quantified using HPLC with a C18 column and a methanol/0.2% formic acid gradient elution program, detecting at 280 nm. The four flavan-3-ols (Epigallocatechin, Catechin, Epigallocatechin gallate, and Epicatechin) were quantified via external calibration curves established with standard solutions ranging from 1 to 50 mg/L. For analysis, wine samples were simply prepared by filtration through a hydrophilic polyethersulfone syringe filter prior to HPLC injection. The analysis of phenolic acids in wine was performed using HPLC with a methanol-acetic acid-water and methanol gradient elution program, detecting compounds at either 280 nm or 320 nm. Prior to injection, the wine samples underwent a liquid–liquid extraction with ethyl acetate, which was then concentrated to dryness under reduced pressure and reconstituted in methanol. The quantification of seven phenolic acids was achieved using an external standard method with calibration curves established for each compound [25].

### 2.4. Volatile Compound Analysis via HS–SPME–GC–MS

Volatile compounds in wine samples were extracted using headspace solid-phase microextraction (HS-SPME). Each 3 mL wine sample was placed in a 10 mL headspace vial with 0.5 g NaCl and 2 μL of 2-octanol internal standard (0.822 g/L). A pre-conditioned DVB/CAR/PDMS fiber was exposed to the sample headspace at 50 °C with 500 rpm agitation for 50 min, then desorbed in the GC injector at 250 °C for 5 min. All analyses were performed in triplicate. The GC-MS conditions included a SUPELCOWAX10 column (60 m × 0.25 mm, 0.25 μm), helium carrier gas at 1.2 mL/min, splitless injection at 250 °C, and a temperature program from 50 °C (held for 1 min) to 180 °C at 3 °C/min, and then to 230 °C at 20 °C/min (held for 15 min) [26]. The MS conditions were as follows: transfer line temperature, 280 °C; ion source temperature, 230 °C; EI ionization; and scan range of 40–450 amu. Qualitative analysis was performed by matching the mass spectra with the NIST library and literature, retaining compounds with SI > 600 that were detected in all the replicates. 2-octanol [27] was added as the internal standard. The relative content of aroma compounds was determined by full-ion scan spectra compared with NIST 2011 and literature and calculated as the average of three replicates.

### 2.5. Sensory Evaluation

To characterize the distinctive flavor profiles of wines produced from different grape varieties, we conducted a standardized sensory analysis employing trained panelists. A nine-member expert panel (4 male, 5 female) evaluated the wines following the International WSET Wine Tasting Guide methodology. The evaluation employed a structured 100-point scoring system assessing (1) visual characteristics (20 points: color depth and clarity), (2) olfactory qualities (30 points: intensity, aromatic harmony, freshness, and complexity), and (3) palate attributes (50 points: body weight, structural balance, and persistence). Additionally, a 5-point intensity scale was used for specific aroma descriptors. All samples were presented in randomized order under controlled tasting conditions (ISO 3591:1977 standard tasting environment) to eliminate serving-order bias, followed by preference ranking assessment. Each treatment group was evaluated in triplicate tasting sessions to ensure result reliability.

### 2.6. Statistical Analysis and Data Processing

Significant differences were analyzed using IBM SPSS Statistics 26 software with analysis of variance (ANOVA). *n* = 3, *p* < 0.05 indicates a significant difference. The results are expressed as mean ± standard deviation (Mean ± SD). Bar charts were drawn using GraphPad Prism 9.0 software. Heat maps, box plots, score plots, and flavor radar plots were generated using Origin 2025 software.

## 3. Results and Discussion

### 3.1. Impact of Yeast Strains on the Fermentation Dynamics of Must

During alcoholic fermentation, yeast converts sugar into ethanol and carbon dioxide while also generating numerous secondary metabolites that significantly impact wine aroma, mouthfeel, and overall quality [28]. In this study, fermentation progress was monitored by measuring soluble solid content (expressed as °Brix).

**Figure 1:**
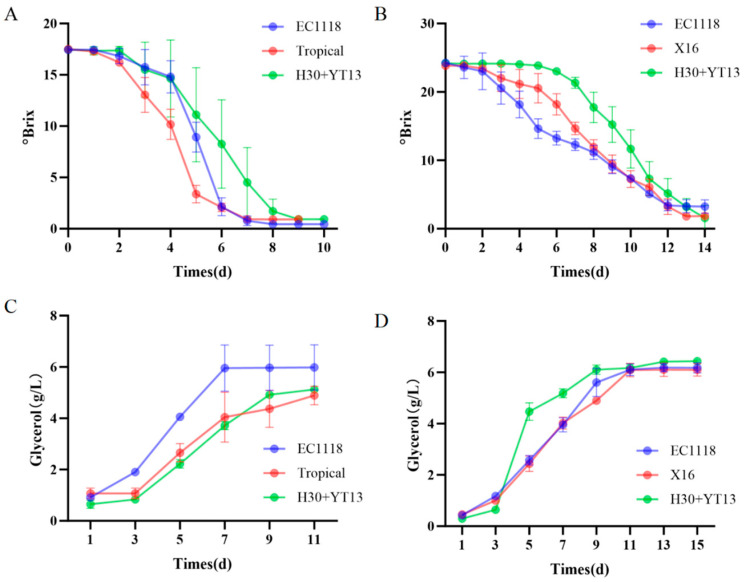
Changes of sugar and glycerol content in Italian Riesling and Petit Manseng during fermentation process: (**A**) Sugar content of Italian Riesling; (**B**) Sugar content of Petit Manseng; (**C**) Glycerol content of Italian Riesling; (**D**) Glycerol content of Petit Manseng.

As shown in Figure 1A,B, the Italian Riesling group completed fermentation within 10 days, whereas Petit Manseng—with higher initial sugar content—required 14 days for full fermentation. The fermentation kinetics differed markedly between inoculation strategies. The single-strain commercial yeast groups initiated a rapid and steady decline in °Brix from the outset. In contrast, the sequential inoculation group (H30 + YT13) exhibited a distinct two-phase profile: a slower initial Brix reduction during the first 4 days of fermentation with *Hanseniaspora uvarum* H30 alone, followed by a pronounced acceleration immediately after the inoculation of *Saccharomyces cerevisiae* YT13. Notably, Brix declined slowly during the first 4 days but dropped rapidly after YT13 inoculation, demonstrating that yeast selection directly modulates fermentation kinetics.

Figure 1C,D reveal that glycerol production surged earlier in commercial yeast-inoculated groups. A sharp glycerol increase occurred on day 5 in sequential inoculation groups, coinciding with YT13 addition. Glycerol is synthesized via the glycolytic (EMP) pathway, and its spike aligns with the metabolic shift triggered by *S. cerevisiae* [29]. Post-fermentation, Petit Manseng wines showed marginally higher glycerol than Italian Riesling, potentially due to elevated initial sugar levels. Fermentation rate and glycerol production vary significantly with yeast strains, reflecting differences in metabolic activation and sugar utilization efficiency. These findings highlight how yeast selection shapes fermentation dynamics and ultimately influence the quality of white wines.

The observed differences in fermentation kinetics and glycerol production underscore the critical role of yeast selection in shaping the fermentation process and wine composition. The accelerated Brix reduction following sequential inoculation with the indigenous strains H30 and YT13 suggests a synergistic effect, where the initial activity of H30 may create a favorable microenvironment for the subsequent dominance of YT13, thereby enhancing fermentation efficiency. The delayed yet sharp increase in glycerol upon YT13 addition aligns with the metabolic shift associated with *S. cerevisiae* activation, highlighting how staged inoculation can strategically modulate metabolite synthesis. Given that glycerol contributes to wine body and smoothness [30], its strain-dependent production pattern further emphasizes the potential of using native yeast combinations to not only guide fermentation dynamics but also influence key sensory attributes. These results collectively indicate that the targeted use of indigenous Yantai yeasts, such as H30 and YT13, offers a viable approach to optimizing both the process and product quality for regional white wines.

### 3.2. Physicochemical Parameters of the Wines

From Table 2, the three fermentation groups from the same grape showed no significant differences in alcohol content, pH value, or volatile acidity. Organic acids play a crucial role in determining the acidity of white wines and are involved in the chemical equilibrium of both wine and grape juice [31]. The primary organic acids include tartaric acid, succinic acid, lactic acid, and citric acid, making their quantification in wines essential.

In the Italian Riesling white wines, the Tropical yeast-inoculated group showed the highest succinic acid content (1.99 ± 0.10 g/L), which was significantly higher than the EC1118-inoculated group. However, no significant differences were found in tartaric acid, citric acid, and lactic acid contents among Italian Riesling wines fermented with different yeast strains. For Petit Manseng white wines, the X16-inoculated group exhibited significantly higher succinic acid content (4.55 ± 2.10 g/L) compared to both EC1118 and H30 + YT13 groups. Tartaric acid was the most abundant organic acid in these wines, with the EC1118-fermented Petit Manseng showing significantly higher tartaric acid content (19.40 ± 1.70 g/L) than other groups. The sequential fermentation group H30 + YT13 demonstrated significantly higher lactic acid content than commercial wine yeast strains, though no advantages were observed in other organic acid concentrations.

It is noteworthy that the composition and concentration of organic acids directly influence the acidity framework, taste balance, and aromatic expression of wine [32]. In this study, the organic acid profile of Italian Riesling remained relatively stable across different yeast treatments; however, the well-balanced acid composition observed in the H30 + YT13 sequential inoculation group likely provided a more stable medium for the expression of other volatile aroma compounds (such as esters and terpenes), thereby contributing to its outstanding floral and fruity intensity in the sensory evaluation. For Petit Manseng wines, the higher tartaric acid in the EC1118 group and the unusually high succinic acid in the X16 group might result in sharper acidity or enhanced bitterness, whereas the increased lactic acid content in the H30 + YT13 group contributed to a softer and rounder mouthfeel [33]. This smoother acidic background synergized with its significantly enhanced ester-driven aromas (particularly tropical fruit notes), collectively shaping a more layered and fruit-dominated sensory profile for this group, even though its total acid content was not the highest.

In conclusion, yeast selection not only determined the absolute concentrations of organic acids but also regulated their overall composition, thereby profoundly influencing the sensory style of the wines. The application of the indigenous yeast combination H30 + YT13 demonstrated potential in optimizing acid structure and enhancing aromatic synergy in both wines. For Italian Riesling, it maintained acid balance and stability, providing an ideal condition for the expression of typical varietal aromas; for Petit Manseng, it softened the wine body through an increased lactate proportion, allowing its intense tropical fruit aromas to be more fully expressed. This indicates that the use of indigenous yeasts for fermentation control is an effective strategy for influencing the flavor structure and typicity of wine through underlying metabolic pathways.

### 3.3. Phenolic Content of White Wines

**Figure 2:**
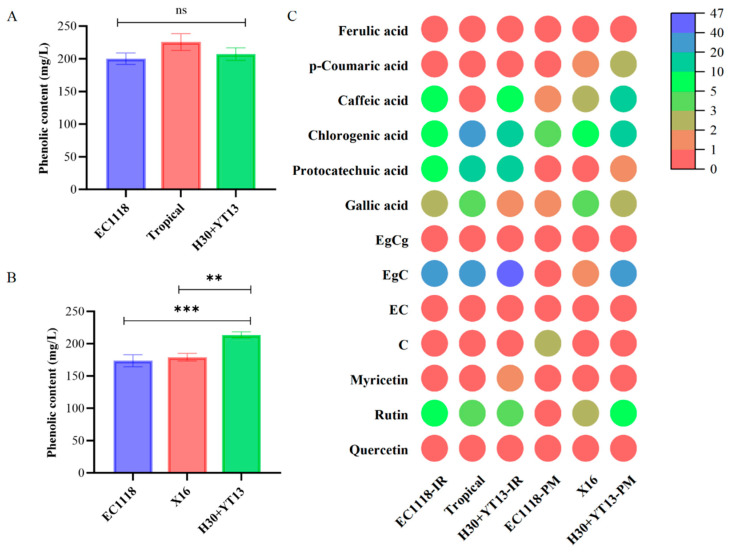
Phenolic compounds of white wines fermented by different yeasts: (**A**) Total phenolic content of Italian Riesling; (**B**) Total phenolic content of Petit Manseng; (**C**) flavonols, flavan-3-ols, and phenolic acid monomers in white wines. (-IR stands for the Italian Riesling group; -PM stands for the Petit Manseng group). Note: **, *** indicate significant differences at the 0.01, and 0.001 levels, respectively; ns indicates no significant difference (*p* > 0.05).

Phenolic compounds not only possess health benefits but also influence the flavor and color of wine [34]. Therefore, determining the total phenolic content is of great significance for evaluating wine quality. Figure 2A,B show the total phenolic contents in white wines made from Italian Riesling and Petit Manseng, respectively, fermented with different yeast strains. In Italian Riesling, the group inoculated with the commercial yeast Tropical exhibited a slightly higher total phenolic content, though no significant differences were observed among the three groups. In contrast, for Petit Manseng, the wines fermented with wild yeast strains H30 and YT13 showed slightly higher total phenolic contents compared to the other two groups, reaching 213.5 ± 4.78 mg/L.

An analysis was conducted on the content of flavonols, flavan-3-ols, and phenolic acid monomers in the wines (Figure 2C). Flavonols and their glycosides are important components in wine, influencing its color, taste, and health-related properties [35]. In the Italian Riesling white wine, different inoculation methods showed no significant effect on flavonol content. However, in the Petit Manseng wine, the H30 + YT13 co-inoculation group exhibited a significantly higher quercetin content of 0.86 ± 0.14 mg/L compared to the other groups. Quercetin contributes to wine’s antioxidant capacity and bitterness and may also enhance color stability through copigmentation. Additionally, the rutin content in this group was 6.85 ± 1.70 mg/L, also significantly higher than in the other treatments. Rutin acts as an antioxidant and may influence astringency and mouthfeel, while also playing a role in protecting against oxidative degradation [36]. Furthermore, myricetin was detected only in the H30 + YT13 inoculated Petit Manseng white wine. Flavan-3-ols are key flavor compounds in wine, known for their antioxidant properties and influence on both color and taste [37]. Epigallocatechin (EgC) is the most abundant flavan-3-ol, contributing to wine’s bitterness, astringency, and potential health benefits [38]. In both Italian Riesling and Petit Manseng white wines, the sequential inoculation group H30 + YT13 showed higher EGC content compared to other groups, which may be attributed to the involvement of non-*Saccharomyces* yeasts leading to increased concentration. Regarding phenolic acid monomers, in the Italian Riesling white wine, the group inoculated with Tropical exhibited the highest gallic acid content after fermentation, reaching 4.30 ± 1.40 mg/L, which was significantly higher than the other two groups, while the levels of other phenolic acid monomers showed little variation. In the Petit Manseng white wine, the co-inoculation group H30 + YT13 showed significantly higher levels of chlorogenic acid and caffeic acid compared to the other groups. Chlorogenic acid contributes to antioxidant activity and may influence bitterness and astringency, while caffeic acid enhances oxidative stability and is associated with antimicrobial effects and potential health benefits.

The distinct phenolic profiles observed, particularly the enhanced levels of specific flavonols and phenolic acids in wines produced with the indigenous H30 + YT13 consortium, underscore the potential of native yeast strains to modulate the phenolic composition and functional properties of white wines. The significant increase in quercetin, rutin, epigallocatechin, chlorogenic acid, and caffeic acid in the H30 + YT13-inoculated Petit Manseng suggests that the enzymatic activities of these yeasts—especially the high β-glucosidase activity of H30—may improve the release and stability of phenolic compounds. This enhancement not only contributes to the wine’s antioxidant capacity and structural complexity (e.g., bitterness and astringency) but may also synergize with the previously reported elevated aroma profiles, collectively enhancing the wine’s overall typicity and regional character.

### 3.4. Volatile Compounds of White Wines

**Figure 3:**
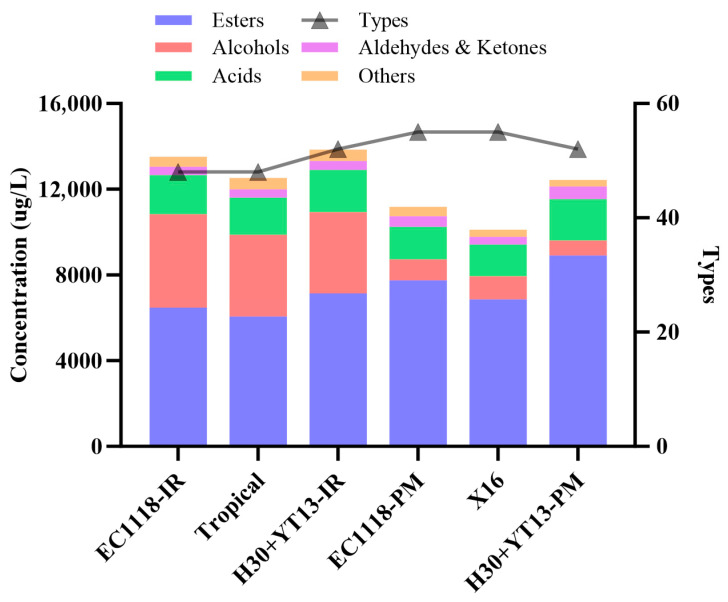
Types and contents of volatile compounds in Italian Riesling and Petit Manseng white wine samples fermented by different yeasts. (-IR stands for the Italian Riesling group; -PM stands for the Petit Manseng group).

Volatile compounds in wine primarily include acids, alcohols, esters, aldehydes, ketones, and terpenes. These volatile components play a crucial role in the balance and harmony of wine aroma, making the measurement of their content an important reference for evaluating wine quality. This study determined the types and concentrations of volatile compounds in Italian Riesling and Petit Manseng white wines fermented with different yeast strains, as shown in Figure 3.

In terms of the number of aroma compounds, differences among the various groups were relatively small. However, with regard to concentration, the volatile compound levels in Italian Riesling white wines were consistently higher than those in Petit Manseng, possibly due to differences in the abundance of aroma precursors. In white wines made from both grape varieties, the co-inoculation groups showed significantly higher overall aroma intensity compared to the single commercial *Saccharomyces cerevisiae* yeast group. This difference was particularly evident in the concentration of esters, which may be attributed to the higher abundance of enzymes catalyzing esterification reactions in non-*Saccharomyces* yeasts.

**Figure 4:**
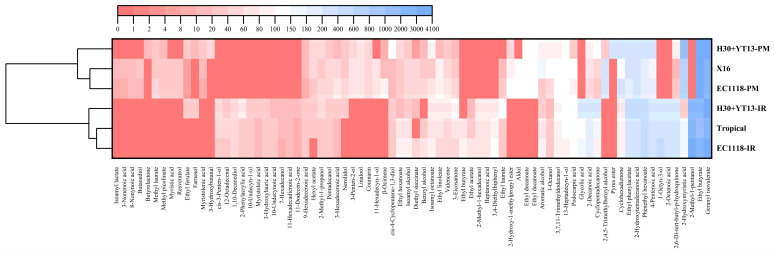
Clustering heatmap visualization of volatile compounds of white wines. Red color represents low concentration, and blue color represents high concentration in the clustered heat map. (-IR stands for the Italian Riesling group; -PM stands for the Petit Manseng group).

Figure 4 presents a heatmap illustrating the specific aroma compounds in the two white wines. In the Italian Riesling white wine, the sequential inoculation group (H30 + YT13) demonstrated notable advantages over the two commercial yeast strains (EC1118 and Tropical) in several aspects. This superiority was particularly evident in the ester compounds, with the sequential inoculation group exhibiting the highest total ester content (7145.75 µg/L). Specifically, key esters such as ethyl butyrate (2338.47 µg/L), phenethyl hexanoate (507.96 µg/L), and geranyl isovalerate (3573.19 µg/L) were present at significantly higher concentrations in the H30 + YT13 group compared to at least one of the commercial yeast groups. These esters typically contribute fruity and floral aromas to wine. Owing to the high β-glucosidase activity of the wild yeast H30, it effectively hydrolyzes aroma precursors in grape berries, releasing free-form terpene aromas. The data clearly show that two important terpenoid compounds, farnesol (25.37 ± 0.61 µg/L) and nerolidol (32.05 ± 4.02 µg/L), were detected exclusively in the H30 + YT13 sequential inoculation group. This result directly demonstrates the important role of non-*Saccharomyces* yeast with high β-glucosidase production in enhancing the distinctive floral and fruity aromas of wine.

Analysis of the volatile compounds in Petit Manseng white wine revealed that sequential inoculation most notably enhanced the ester profile. The total ester content in the H30 + YT13 group (8918.01 µg/L) substantially exceeded that of the two control groups. Key esters contributing importantly to the wine’s fruity aroma, including phenethyl acetate (303.26 µg/L), phenethyl hexanoate (258.29 µg/L), and geranyl isovalerate (3396.74 µg/L), were present at significantly higher concentrations in the sequential inoculation group. The analysis of aldehydes and ketones showed that the H30 + YT13 group also had the highest total content in this category (585.5 µg/L), with significantly higher levels of compounds such as cyclopentadecanone (113.53 µg/L) compared to the other groups. Cyclopentadecanone typically imparts a musky note [39]. Their pronounced presence considerably enhances the overall aroma complexity and depth. Most critically, the specific accumulation of terpenoid compounds can be directly attributed to the high β-glucosidase activity of the wild yeast H30. The data clearly indicate that farnesol (13.54 ± 0.24 µg/L) was detected exclusively in the H30 + YT13 group. Meanwhile, although linalool was identified in all three groups, its concentration remained the highest (70.44 µg/L) in the sequential inoculation group. These terpenes are primary sources of elegant floral, rose, and citrus aromas in wine. This finding robustly demonstrates that sequential inoculation effectively hydrolyzes bound aroma precursors into free volatile terpenes, significantly enhancing the varietal typicity of Petit Manseng white wine.

The comprehensive analysis indicates that sequential inoculation (H30 + YT13) was effective for both varieties, but its impact was more significant and critical for Italian Riesling. This is because the strategy not only resulted in the largest relative increase in total volatile compounds (+15%) in Italian Riesling but also specifically synthesized valuable terpenes (farnesol and nerolidol) that were exclusive to this group, representing a qualitative “from nothing to something” transformation. In contrast, its effect on Petit Manseng was primarily a quantitative “from limited to substantial” increase in esters. Therefore, sequential inoculation, particularly using yeast with high β-glucosidase activity, offers an important advantage for enhancing the distinct varietal character of aromatic varieties like Italian Riesling.

### 3.5. Sensory Evaluation

**Figure 5:**
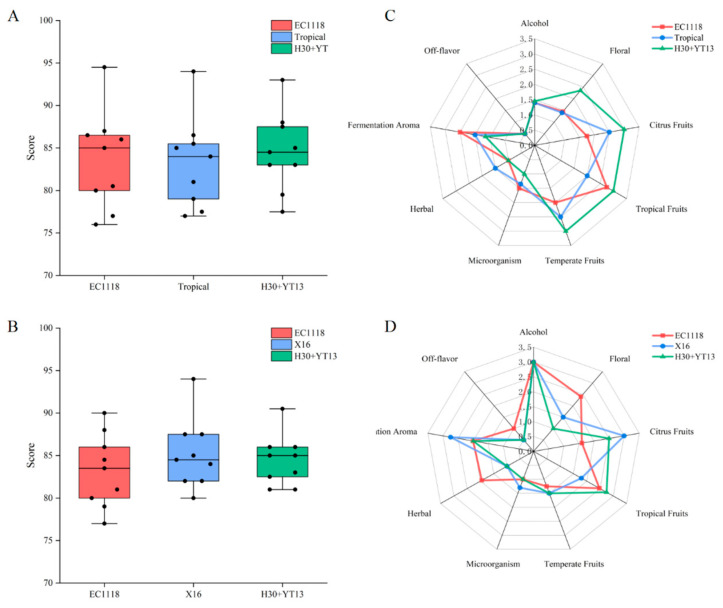
The box plot of scores and flavor radar chart of different white: (**A**) Box plot of Italian Riesling; (**B**) Box plot of Petit Manseng; (**C**) Flavor radar chart of Italian Riesling; (**D**) Flavor radar chart of Petit Manseng.

Figure 5A,C present the box plot of scores and flavor radar chart for Italian Riesling. In the Italian Riesling white wine, the H30 + YT13 group demonstrated a clear advantage in almost all positive aroma attributes. Its intensity of Floral (2.35), Citrus Fruits (3), and Temperate Fruits (3) far surpassed that of the commercial yeast groups. This finding perfectly aligns with the exceptionally high ester levels and unique terpenes detected in the volatile compound analysis, providing strong evidence for the crucial role of H30’s β-glucosidase activity in unlocking varietal-specific aromas. Concurrently, the group exhibited a significantly lower level of undesirable Fermentation Aroma (1.65), resulting in a purer and more elegant nose. Consequently, it achieved the highest average sensory score (85.7), consistently receiving t0068e top ratings from the panel.

In contrast, the impact of sequential inoculation on Petit Manseng was different. Figure 5B,D present the box plot of scores and flavor radar chart for Petit Manseng. Its advantages were primarily reflected in enhanced Tropical Fruits (2.75) and Fermentation Aroma (2) intensity, likely stemming from its vastly elevated ester production. However, its Floral and Citrus Fruits scores were lower than those of some commercial yeast groups, possibly due to differences in the variety’s aroma precursors. Although the final overall scores of the three groups were very close, the H30 + YT13 group developed an aromatic profile with a more intense tropical style.

Sequential inoculation H30 + YT13 is a highly effective technique for improving the sensory quality of wine, but its outcomes are variety-dependent. It offers a revolutionary improvement for aromatic varieties like Italian Riesling, greatly enhancing their varietal typicity. For Petit Manseng, it effectively optimizes the aromatic structure, imparting a more pronounced fruity character.

## 4. Conclusions

This study comprehensively evaluated the effects of different yeast fermentations on the overall quality of Italian Riesling and Petit Manseng white wines from the Yantai region. The results demonstrated that sequential inoculation with indigenous non-*Saccharomyces* yeast H30 and *Saccharomyces cerevisiae* YT13 not only modified fermentation kinetics but also significantly influenced the wines’ physicochemical composition, phenolic content, and aromatic profile. The process slightly increased the total phenolic content in Petit Manseng wines and significantly enhanced the concentration of specific phenolics (e.g., quercetin, rutin, and caffeic acid), thereby improving their antioxidant potential. Regarding volatile compounds, sequential inoculation notably elevated total ester levels and specifically released unique terpenes (such as farnesol and nerolidol) in Italian Riesling wines, which were undetectable in wines produced with commercial yeasts. These physicochemical and aromatic improvements were corroborated by sensory evaluations, with sequentially inoculated wines receiving higher scores for floral and fruity attributes and exhibiting superior overall balance. This study confirms that sequential inoculation can holistically enhance the chemical composition and sensory quality of white wines in a variety-dependent manner, offering valuable insights for developing regional typicity in Yantai white wines.

## Figures and Tables

**Table 1:** Fermentation groups.

Grape Variety	Fermentation Groups
Italian Riesling	EC1118
	Tropical
	H30 + YT13
Petit Manseng	EC1118
	X16
	H30 + YT13

**Table 2:** Basic physicochemical parameters of fermented white wines.

Grape Variety	Yeast	Basic Physicochemical Parameters								
		Residual Sugar (g/L)	Alcohol Content% (vol)	pH Value	Volatile Acids (g/L)	Malic Acid (g/L)	Succinic Acid (g/L)	Tartaric Acid (g/L)	Citric Acid (g/L)	Lactic Acid (g/L)
Italian Riesling	EC1118	3.96 ± 0.11 a	9.90 ± 0.10 a	2.96 ± 0.02 a	0.48 ± 0.25 a	2.07 ± 0.05 a	1.12 ± 0.43 b	13.79 ± 1.53 a	0.86 ± 0.17 a	0.77 ± 0.04 a
	Tropical	3.76 ± 0.20 a	9.93 ± 0.05 a	2.86 ± 0.10 a	0.21 ± 0.10 a	1.86 ± 0.15 a	1.99 ± 0.10 a	12.74 ± 2.80 a	0.90 ± 0.20 a	0.95 ± 0.06 a
	H30 + YT13	3.23 ± 0.25 a	9.80 ± 0.43 a	2.94 ± 0.05 a	0.23 ± 0.00 a	2.23 ± 0.47 a	1.48 ± 0.13 ab	11.40 ± 1.98 a	0.70 ± 0.01 a	0.71 ± 0.11 a
Petit Manseng	EC1118	3.90 ± 1.31 a	13.37 ± 0.40 a	2.89 ± 0.11 a	0.93 ± 0.06 a	3.80 ± 0.17 a	0.69 ± 0.15 b	19.40 ± 1.72 a	0.62 ± 0.24 a	0.30 ± 0.05 b
	X16	3.43 ± 1.38 a	13.10 ± 0.45 a	2.88 ± 0.10 a	0.96 ± 0.17 a	3.33 ± 0.20 ab	4.55 ± 2.17 a	16.01 ± 1.17 b	0.35 ± 0.01 a	0.60 ± 0.18 ab
	H30 + YT13	3.13 ± 1.54 a	12.57 ± 1.72 a	2.85 ± 0.03 a	0.84 ± 0.08 a	3.00 ± 0.26 b	0.67 ± 0.06 b	15.67 ± 0.51 b	0.34 ± 0.05 a	0.83 ± 0.11 a

Note: Data are the mean of three replicate fermentations ± SD. Values within the same row followed by a common letter are not significantly different according to Tukey’s HSD test (*p* < 0.05).

## Data Availability

The data that support the findings of this study are available from the corresponding author upon reasonable request.
